# Modular approach to customise sample preparation procedures for viral metagenomics: a reproducible protocol for virome analysis

**DOI:** 10.1038/srep16532

**Published:** 2015-11-12

**Authors:** Nádia Conceição-Neto, Mark Zeller, Hanne Lefrère, Pieter De Bruyn, Leen Beller, Ward Deboutte, Claude Kwe Yinda, Rob Lavigne, Piet Maes, Marc Van Ranst, Elisabeth Heylen, Jelle Matthijnssens

**Affiliations:** 1 KU Leuven - University of Leuven, Department of Microbiology and Immunology, Laboratory of Viral Metagenomics, Rega Institute for Medical Research Leuven, Belgium; 2 KU Leuven - University of Leuven, Department of Microbiology and Immunology, Laboratory for Clinical Virology, Rega Institute for Medical Research Leuven, Belgium; 3 KU Leuven - University of Leuven, Department of Biosystems, Laboratory of Gene Technology, Faculty of Bioscience Engineering, Belgium

## Abstract

A major limitation for better understanding the role of the human gut virome in health and disease is the lack of validated methods that allow high throughput virome analysis. To overcome this, we evaluated the quantitative effect of homogenisation, centrifugation, filtration, chloroform treatment and random amplification on a mock-virome (containing nine highly diverse viruses) and a bacterial mock-community (containing four faecal bacterial species) using quantitative PCR and next-generation sequencing. This resulted in an optimised protocol that was able to recover all viruses present in the mock-virome and strongly alters the ratio of viral versus bacterial and 16S rRNA genetic material in favour of viruses (from 43.2% to 96.7% viral reads and from 47.6% to 0.19% bacterial reads). Furthermore, our study indicated that most of the currently used virome protocols, using small filter pores and/or stringent centrifugation conditions may have largely overlooked large viruses present in viromes. We propose NetoVIR (Novel enrichment technique of VIRomes), which allows for a fast, reproducible and high throughput sample preparation for viral metagenomics studies, introducing minimal bias. This procedure is optimised mainly for faecal samples, but with appropriate concentration steps can also be used for other sample types with lower initial viral loads.

The sum of the genomes present in the human microbiota living in and on us, including bacteria, archaea, viruses, parasites and fungi, is referred to as the human microbiome^1^. In the last decade, great efforts have been made to study the human microbiome, the microbiome in oceans, soil or animals^2^,^3^,^4^. However, the great majority of these studies focused on bacteria, targeting their entire genomes using a shotgun approach or targeting specific regions such as conserved bacterial 16S ribosomal RNAs^5^,^6^. Although shotgun sequencing provides opportunities to analyse all microbial DNA, the larger average genome size of bacteria compared to viral genomes complicates a detailed analysis of the virome. In addition, these approaches usually overlook viral RNA genomes present in the microbiome^7^. As such, the human virome represents the viral component of the human microbiome, which is its most ubiquitous and genetically diverse fraction. The virome includes viruses infecting the host, viruses infecting eukaryotes present in the microbiota, viruses infecting prokaryotes present the microbiota (bacteriophages) and viruses infecting neither of them (e.g. plant viruses in the gut)^8^.

Next generation sequencing (NGS) has revolutionised the discovery of novel viruses in humans and animals in various ecosystems^9^,^10^,^11^. In contrast, the role of the virome in complex human disease has been less well characterised, even though efforts have been made recently to study the role of viruses in Inflammatory Bowel Disease, diabetes, Acquired Immune Deficiency Syndrome and transplant patients^12^,^13^,^14^,^15^. A major limitation in better understanding the role of the human gut virome in health and disease is the lack of validated methods allowing for high throughput and reproducible virome analyses.

Usually, the majority of genetic material in a sample is of non-viral origin, which makes accurately studying the virome more challenging than studying other components of the microbiome^16^. Viruses also lack universally conserved genomic regions and are both genetically and morphologically highly diverse^8^. Key-steps for studying viromes are the enrichment for virus-like-particles (VLPs) from a sample and the performance of random amplification, if the starting material needs to be increased before NGS library preparation^16^. These steps should preferentially avoid losses of any types of viruses and minimise bias introduced during sample preparation steps. However, this is challenging as viruses are highly diverse and might be removed during particular steps of VLP enrichment.

Several studies have attempted to quantify the efficiency of their virome preparation protocols. Firstly, Sachsenröder and colleagues spiked animal faecal samples with three bacteriophages (T4, M13 and MS2). They were able to detect all three viruses using pyrosequencing after sample purification, although viral recovery rates were highly variable^17^. However, no bacterial or rRNA removal efficiency was tested and only one VLP purification condition was evaluated. Hall and colleagues tested a limited number of combinations of centrifugation, filtration and nuclease treatment, using low concentrations of three medium-small sized viruses (adenovirus, influenza A and human enterovirus), bacterial and human cells^18^, showing that different methods had a significant impact on virus recovery. Li and colleagues pooled samples (allantoic fluid, cell culture and faecal material) containing twenty five human viruses to compare different purification and amplification methods^19^. Their protocol was not able to recover all viruses and showed that recovery of viral reads was highly diverse when different sample preparation methods were used. Kohl and colleagues used four viruses (vaccinia virus, orthoreovirus, influenza virus and Sendai virus) to optimise a protocol to recover viruses from tissue. They used quantitative PCR (qPCR) assays to detect relative losses of viruses after different purification and amplification procedures, again showing that the use of different methods has a strong effect on the final sequence outcome^20^. Recently, Kleiner and colleagues developed an artificial microbiome consisting of six bacteriophages (P22, T3, T7, ϕ6, M13 and ϕVPE25) and two bacteria (*Listeria monocytogenes* and *Bacteroides thetaiotaomicron*). However, recovery of the dsRNA phage was not successful and recovery of viruses highly depended on the combination of methods used^21^. Finally, Rosseel and colleagues spiked Newcastle disease virus in serum and tissue and tested for filtration, DNase treatment and rRNA removal. This protocol was only tested for a single virus and can only be used for RNA viruses^22^.

Although these studies provide valuable information, most of them only compare final outcomes of their procedures, do not take bacteria into account, or use limited sets of viruses in their studies. Our study individually evaluated the performance of each step of the procedure and systematically studied their effect on a unique mock-virome an artificial bacterial community, as well as on 16S rRNA to investigate bias introduced during each step and to obtain the most favourable virus-to-bacteria ratio. In conclusion, a detailed and reproducible protocol is proposed for viral metagenomics (Fig. 1 and Protocol S1), although initial concentration steps might be required for samples with low initial viral load such as soil or ocean water samples. In addition, the modular approach of our study allows researchers to customise sample preparation depending on their needs or particular virus(es) of interest.

## Results

A mock-virome (containing nine highly diverse viruses/phages), as well as a bacterial mock-community (containing four bacterial species common in the gut) were assembled to determine the effect of various sample treatment procedures and allowed to select for procedures that: 1) recover all viruses present in a sample, 2) alter the ratio of viral/(bacterial + rRNA) genetic material in favour of the viruses, and 3) introduce the least amount of bias in the relative distribution of viruses in a sample. Homogenisation, centrifugation, chloroform treatment, filtration and random amplification of nucleic acids were tested on the individual viral and bacterial communities to obtain a clear and clean understanding of the effect of the different tested procedures, avoiding unexpected interactions of viruses and bacteria.

### Homogenisation with ceramic beads reduces number of viral particles

To obtain reproducible results a proper homogenisation of biological samples (such as tissue or faecal samples) is crucial and minimises subsequent filter clogging^23^. As benchtop vortexers do not provide a standardised approach for sample homogenisation, a tissue homogeniser was used. The mock-virome and bacterial mock-community were subjected to homogenisation at different speeds (3000 and 5000 rpm) and with or without beads of different sizes (Ø0.1 and Ø2.8 mm) and compared to a non-homogenised control (Fig. 2). Homogenisation with Ø2.8 mm beads led to a destruction of viral particles irrespective of homogenisation speed. The reduction was largest for coronavirus (99.5% and 99.6% and Ct differences of 8.5 and 8.9 for 5000 and 3000 rpm, respectively) and mimivirus (96.0% and 97.7% and Ct differences of 6.0 and 6.3 for 5000 and 3000 rpm, respectively). In addition, Ø2.8 mm beads led to a 316% and 350% increase in the detection of 16S rRNA at 5000 and 3000 rpm, respectively (Ct difference 2.3 and 2.4, respectively). For the bacteria, Ø2.8 mm beads also showed increases ranging from 612% (3.2Ct decrease) for the *Bacteroides* to 6184% for the *E.coli* (6.1Ct decrease). Homogenisation at 5000 rpm (without beads or with Ø0.1 mm beads) showed a larger reduction in viral particles than homogenisation at 3000 rpm. Reduction of viral particles was lowest using 3000 rpm homogenisation without beads (the maximum percentage of reduction was 59.6% for the pepino mosaic virus, 1.07Ct), and did not increase the amount of bacterial DNA/rRNA. All conditions resulted in well-homogenised suspensions suitable for subsequent filtration experiments (data not shown).

### Centrifugation conditions strongly influence the reduction in bacterial and viral particles

Centrifugation is commonly used to precipitate larger particles, such as cells and cellular debris, while viruses remain in solution. Since this step differs greatly among studies^24^,^25^,^26^, we decided to test a mild and medium centrifugal force (100 *g* and 17000 *g*, respectively) as well as short (3 min) and medium (30 min) centrifugation times on our mock-communities (Fig. 3). Higher *g*-forces or longer centrifugation times were not tested as this would result in even higher viral losses. Centrifugation at 100 *g* for 3 min showed limited differences compared to the control for viruses, bacteria and rRNA. Only for *Lactobacillus* a reduction of more than 1 Ct was observed (68.6%; 1.8Ct increase). Centrifugation for 30 min at 100 *g*, resulted in an increased reduction of mimivirus (94.6%; 4.5Ct). For the bacteria, the highest reductions were observed for the *Lactobacillus* (99.0%; 7Ct) and *Bifidobacterium* (96.0%; 5Ct), whereas *E.coli* and *Bacteroides* showed less pronounced reductions (80.6% or 2.4 Ct and 48% or 0.95 Ct, respectively). Centrifugation at 17000 *g* for both 3 and 30 min resulted in more than 99.99% reductions for *Bifidobacterium*, *E.coli* and *Bacteroides*. The reduction in *Lactobacillus* was slightly lower (99.7%–99.8%), whereas the reduction in the rRNA was 98.5–99.4%. For 3 minutes at 17000 *g* the losses for viruses were negligible, except for polyomavirus (43.3% loss, 1.2Ct), herpesvirus (65.3% loss, 1.3Ct), and especially mimivirus (96.9% loss, 5.4Ct). These findings were much more pronounced when centrifugation was performed at 17000 *g* for 30 minutes. Surprisingly, more than 90% reduction was observed for polyomavirus, coronavirus and herpesvirus, and 99.9% for mimivirus. Although the samples where thoroughly homogenised, it cannot be excluded that this observation is due to viral aggregates still present in the sample.

### Filtration: keep the viruses or get rid of bacteria?

Filtration is an efficient and widely used method to enrich viral particles by removing bacterial and host cells (human/animal/plant). Therefore, the mock-virome and bacterial mock-community were filtered with a 0.8-μm centrifugal filter (PES), a 0.8-μm polycarbonate filter (PC), a 0.45-μm centrifugal filter (PVDF) or a 0.22-μm centrifugal filter (PVDF) and compared with an unfiltered control (Fig. 4). Filtration with the two 0.8-μm and 0.45-μm filters showed limited effects on most of the viruses in the mock-virome, except for mimivirus, the largest virus in the mock-virome, for which a 81.7% (3.8Ct increase), a 95.9% (6.8Ct) and a 99.0% (9.9Ct) reduction was observed for the 0.8-μm PES, the 0.8-μm PC and 0.45-μm filters, respectively. The least mimivirus was recovered using the 0.22-μm filter (99.90% reduction, 13.95Ct), and also 82.0% (4.1Ct) reduction was observed for herpesvirus using the 0.22-μm filter. The use of the 0.8-μm PC filter resulted in a reduction of 99.1% (7.5Ct) and 99.2% (7.9Ct) for *Lactobacillus* and *Bifidobacterium*, respectively, whereas *E.coli* (80.7%, 2.5Ct) and *Bacteroides* (50.1%, 1.04Ct) showed modest reductions. A much more efficient removal of bacteria was obtained with the 0.8-μm PES, 0.45-μm and 0.22-μm filters, removing 99.5% to 99.90% of all bacteria present in the bacterial mock-community. Independent of the filter used, rRNA was less efficiently removed than bacteria (30–75% reduction; 0.7–2.4Ct).

### Chloroform treatment efficiently removes bacteria but alters viral abundances

Chloroform disrupts lipid membranes and is often used to remove bacteria. However, some viruses are enveloped, and their capsid can become unstable after envelope removal. To determine the effects of chloroform, the mock-virome as well as the bacterial mock-community were incubated in the presence of 1%, 5%, 10% and 20% chloroform and compared with a non-chloroform exposed control sample. Incubation in 1% chloroform resulted in a reduction less than 1 Ct for all the enveloped and non-enveloped viruses, except for mimivirus showing a moderate loss of 53.2% (1.3Ct increase) (Fig. 5A). When incubating with higher percentages of chloroform (5–20%), no or very limited losses for circovirus, parvovirus, pepino mosaic virus and LIMEstone virus were observed. The highest reductions were observed for the enveloped coronavirus (range of reduction: 81.4–89.9%; 2.6–2.8Ct) and mimivirus (61.4–77%; 1.6–2.5Ct), and as well as for the non-enveloped rotavirus (range of reduction: 74–77%; 2.3–2.5Ct). In contrast, for the enveloped herpesvirus only 18.8–31.2% (0.38–0.69Ct) reduction was observed. Gram-positive bacteria were sensitive to 1% chloroform, resulting in a decrease of 97.6–99.4% (6.1–7.9Ct) for *Bifidobacterium* and *Lactobacillus*, respectively, whereas Gram-negative bacteria were less affected with reductions below 90% (Fig. 5B). However, high concentrations of chloroform (10–20%) resulted in a substantial reduction of all bacterial species (99.6–99.999%; 8.8–17.6Ct). Finally, rRNA was reduced by 83.5–94.9% (2.9–4.8Ct), and only a limited effect was observed when the percentage of chloroform was changed (Fig. 5B).

### Random amplification: the necessary bias that amplifies all viruses

To sequence viral RNA genomes, a reverse transcription step is required to convert RNA to DNA and all single-stranded genomes to double-stranded. Furthermore, the low amount of genetic material from a sample often requires an amplification step. Amplification was performed using an adapted version of the Whole Transcriptome Amplification Kit 2 (WTA2) to amplify both DNA and RNA. To determine bias introduced by amplification, the number of cycles was varied with 5-step intervals between 7 and 22 and compared with a non-amplified control. At 7 amplification cycles an increase of at least 374% (3.7-fold) was observed for all viruses in the mock-virome. A strong increase in the concentration of all viruses was observed at 12 (range 26–808 fold increase) and 17 cycles (range 170–6466 fold increase), whereas at 22 cycles, 3 out of 9 viruses in the mock-virome showed lower yields than at 17 cycles. The same procedure was followed for the bacterial mock-community and bacterial amplification after 7 cycles for *Lactobacillus*, *E.coli* and *Bacteroides* ranged between 1061–2033%, and increased 5611% for rRNA (Fig. 6). An increase between 7844% and 19088% was observed for 12 amplification cycles, whereas rRNA increased to 14920%. For 17 and 22 cycles, the median amplification decreased, and for 22 cycles amplification pattern was similar to 7 cycles. No amplification was observed for the *Bifidobacterium* for 7, 17 and 22 cycles and very limited amplification (7.5%) for 12 cycles.

### The percentage of sequencing reads correlates with number of viral genome copies after amplification

Based on all results above, seven different work-flows were selected including homogenisation at 3000 rpm without beads and 17 amplification cycles, in combination with different conditions of filtration (0.8 PC/PES, 0.45 and 0.22-μm) and/or centrifugation (3 min at 17000 *g*). A combined artificial community of viruses and bacteria was subjected to the selected conditions and sequenced using an Illumina HiSeq2500™. In the control sample sequencing data, 47.6%, 43.2% and 1.6% of the reads were attributed to bacteria, viruses and rRNA, respectively (Fig. 7A and Table S4). The unmapped reads obtained were attributed to residual host and bacterial DNA derived from the preparation of viral stocks, such as *Dickeya solani* and Chlamydiales. The use of the 0.8-μm PC filter resulted only in minor reduction in the percentage of reads mapping to bacteria (47.3%). The remaining filtering and/or centrifugation conditions resulted in a dramatic reduction in the percentage reads that mapped to bacteria (ranging from 0.39% to 0.71%), while the number of viral reads increased to 88.8–96.7%. The 0.8-μm PES filter plus centrifugation condition yielded the highest percentage of viral reads, of which most were attributed to pepino mosaic virus (33.9%), LIMEstone virus (32.9%) and rotavirus (20.6%) (Fig. 7B). When comparing the distribution of the viral reads of the control, the four protocols without centrifugation showed an expansion of the LIMEstone virus reads, mainly at the expense of rotavirus and pepino mosaic virus reads. Also, the combination of centrifugation with filtration showed a minimal increase of LIMEstone virus and pepino mosaic virus reads, but at the expense of parvovirus, polyomavirus and circovirus reads. The percentage of herpesvirus reads in the control was low (0.11%) and slightly increased with centrifugation plus 0.8-μm PES filter (0.18%). For the remaining conditions, herpesvirus reads ranged from 0.04% to 0.24%. The mimivirus viral reads in the control (0.91%) could only be increased when filtering with 0.8-μm PC filter (2.2%), but were strongly reduced when using the other protocols (0.011%–0.034%). Although nearly all bacteria were removed, the distribution of remaining bacterial sequence reads was dominated by *Bacteroides* in the control and 0.8-μm PC filter (41.3% and 43.5% of total reads, respectively), whereas in the remaining conditions *E.coli* was the most abundant (0.14–0.40%) (Fig. 7C and Table S4). The rRNA obtained reads were low for the control (1.59%) and reduced to 0.2%–0.004% for the treated conditions. Overall, a good correlation was observed between the number of viral copies as determined by qPCR and the percentage of mapped reads (R^2^ = 0.91). For most viruses a high correlation between 0.88 and 0.97 was observed, only for circovirus and herpesvirus the correlation was slightly lower (R^2^ = 0.75 and 0.63, respectively) (Fig. 7D). For parvovirus no correlation was observed (R^2^ = 0.16). No correlation for bacteria was calculated as the qPCR measurements for the bacteria after treatment were often below the detection limits of the used assays.

**Figure 1:**
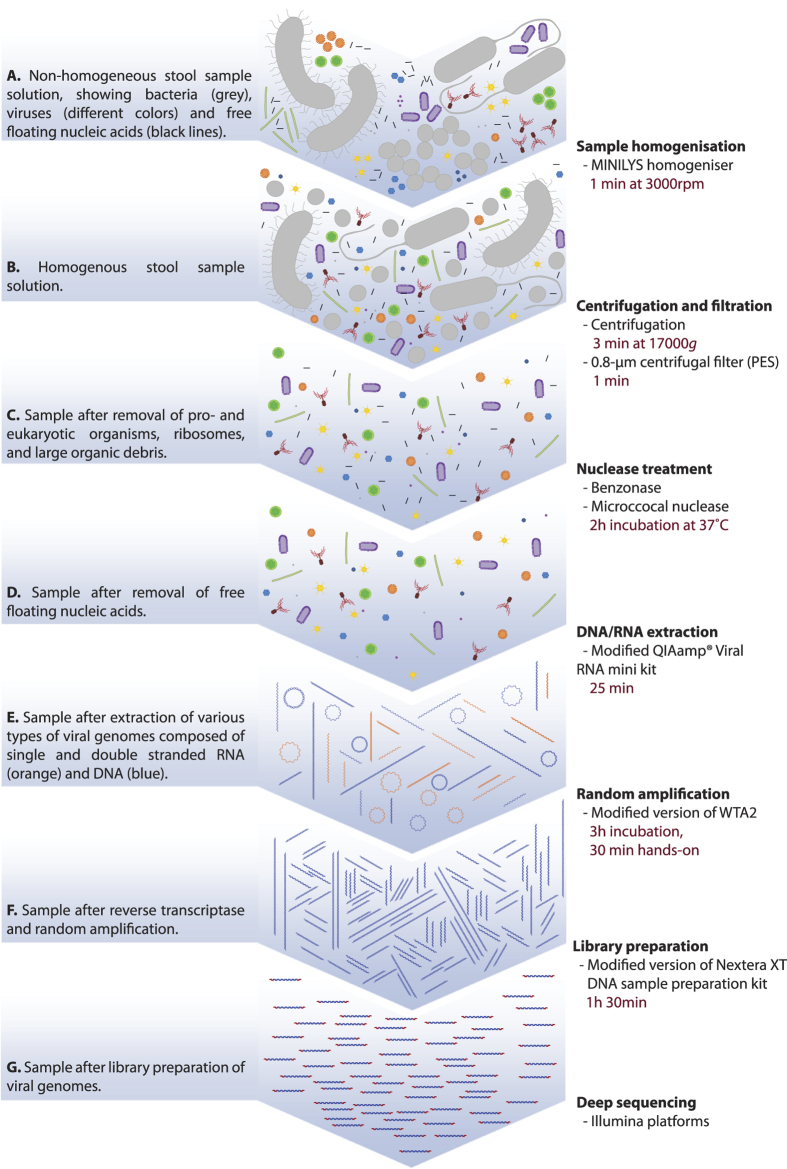
Schematic concise description of the proposed NetoVIR protocol. Estimations of incubation time and total time for each step are shown. On average, the protocol takes 8 h to complete. A detailed protocol is described in Protocol S1 (Supplementary information).

**Figure 2:**
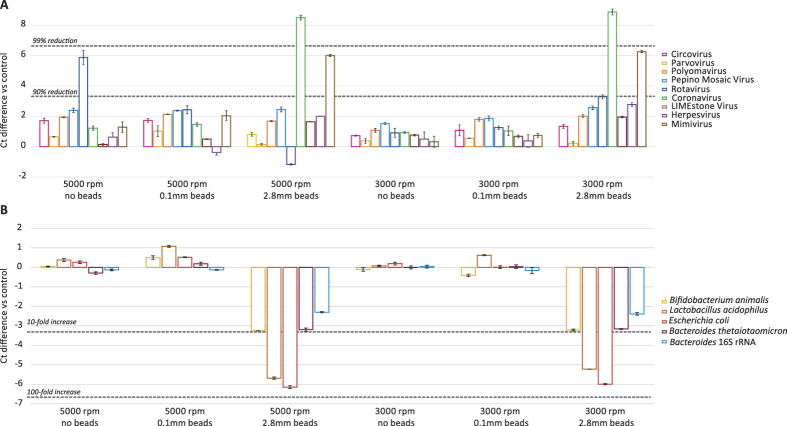
Ct differences vs control for different homogenisation experiments performed on the mock-virome (A) and on the bacterial mock-community and *Bacteroides* 16S rRNA (B). Standard deviations are based on three qPCR replicates.

Taking into account the sequencing results, a favoured protocol named NetoVIR (Novel enrichment technique of VIRomes) was selected. NetoVIR consisted of homogenisation at 3000 rpm for 1 min without beads, centrifugation for 3 min at 17000 *g* plus 0.8-μm PES filter filtration and 17 amplification cycles (Fig. 1), and was further tested for reproducibility. Two replicates were processed in parallel and evaluated before and after the procedure (Fig. S1 and Table S5). Before the procedure, standard deviations ranged from 3% for the LIMEstone virus to 19% for the polyomavirus. After treatment, standard deviations ranged from 3% for the coronavirus to 24% of the circovirus. As a result of a low viral copy number after centrifugation and filtration, a slightly higher standard deviation was observed for mimivirus (38%). Overall, the standard deviation was comparably low before and after the procedure (Fig. S1 and Table S5).

## Discussion

Aberrations of the human virome have been implicated in acute and chronic diseases. In order to systematically investigate virome-disease relationships, reproducible, inexpensive and high-throughput virome methods are urgently needed. In this study we investigated bias that can be introduced during various sample treatment procedures commonly used in viral metagenomics studies on artificial, well-characterised communities of viruses and bacteria. With the results obtained we established a protocol for viral metagenomics that maximises removal of non-viral nucleic acids, while minimising disturbances in viral relative abundances.

A homogeneous solution is crucial for optimal and reproducible viral particle purification from most biological or environmental sample. No or limited homogenisation might be needed for cell culture supernatants, water or blood/serum samples. Faeces, respiratory or soil samples may benefit from a proper homogenisation, whereas tissue samples cannot proceed without a thorough homogenisation for virus release, often using beads of varying sizes. In this study we evaluated the effect of several homogenisation conditions on our artificial communities. Homogenisation with 2.8 mm beads resulted in severe loss of coronavirus and mimivirus, and particularly in a strong increase of bacteria and bacterial rRNA. Since the nucleic acids extraction kit used in this study was not optimised to extract bacterial DNA, extraction efficiency might be enhanced when homogenising with 2.8 mm beads by severely impairing bacterial cell walls. For any virome study this seems to be highly undesirable and should therefore be avoided. If unavoidable, viral losses have to be considered when interpreting the obtained results. The same effect was not observed when 0.1 mm beads were used, suggesting that a bigger mechanical force is necessary to increase extraction efficiency. Overall, the smallest effects on viral losses were observed using the lowest homogenisation speed without beads. In our experience, a proper homogenisation without beads is sufficient to destroy faecal aggregates and obtain a workable suspension.

**Figure 3:**
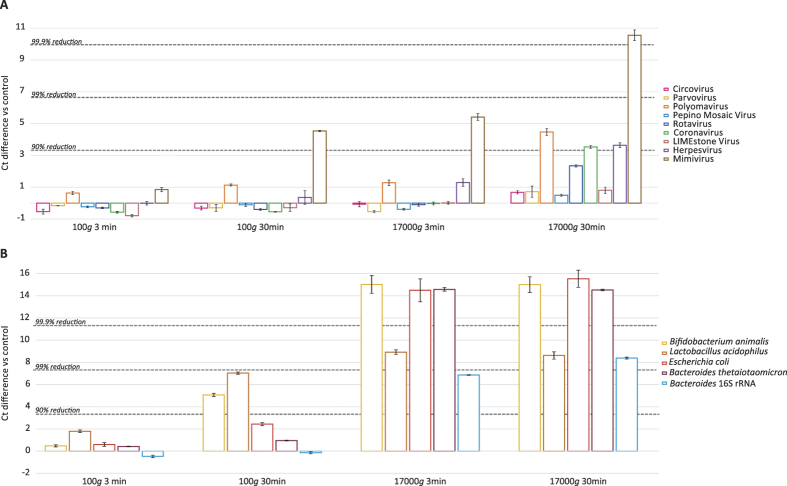
Ct differences vs control for centrifugation conditions tested on the mock-virome (A), on the bacterial mock-community and *Bacteroides* 16S rRNA (B). Standard deviations of the qPCR replicates are displayed.

Centrifugation, filtration and chloroform treatment are commonly used methods to enrich samples for viral particles. The increasing number of reports on giant viruses, which have similar physical dimensions as small bacteria makes this challenging^27^,^28^,^29^. Centrifugation is widely used in virome studies prior to filtration^13^,^14^,^26^, but effects on the virome have been quantitated poorly. Centrifugation for 3 min at 17000 *g* resulted in the removal of almost all bacteria, but unfortunately a significant reduction of mimivirus was also observed (Fig. 3). Interestingly, centrifugation at 17000 *g* also resulted in a decrease around 99% in rRNA despite the fact that ribosomes have a size (20–30 nm) similar to those of many other viruses in our mock-virome (Table 1). However, the higher density of ribosomes (more than 1.6 g/cm^2^ 30) in comparison to viral particles may have led to their more efficient precipitation (Table 1). Alternatively this observation may be partially explained by the fact that some of the ribosomes may still have been attached to larger bacterial cellular components.

**Figure 4:**
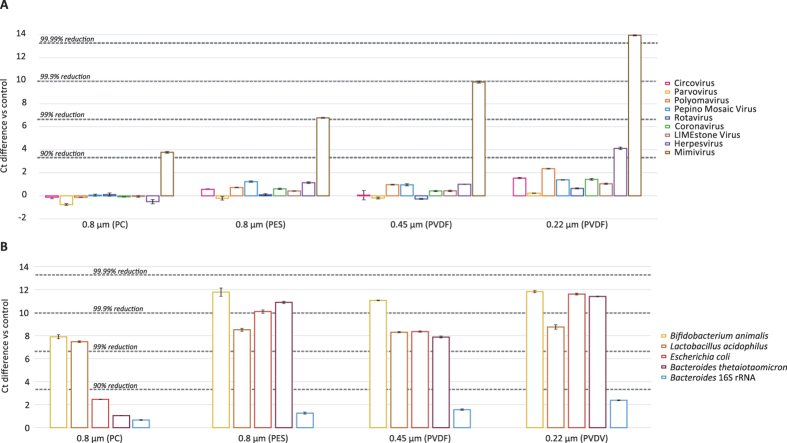
Ct differences vs control for filtration experiments performed on the mock-virome (A), bacterial mock-community and *Bacteroides* 16S rRNA (B). Standard deviations of the qPCR replicates are displayed.

**Figure 5:**
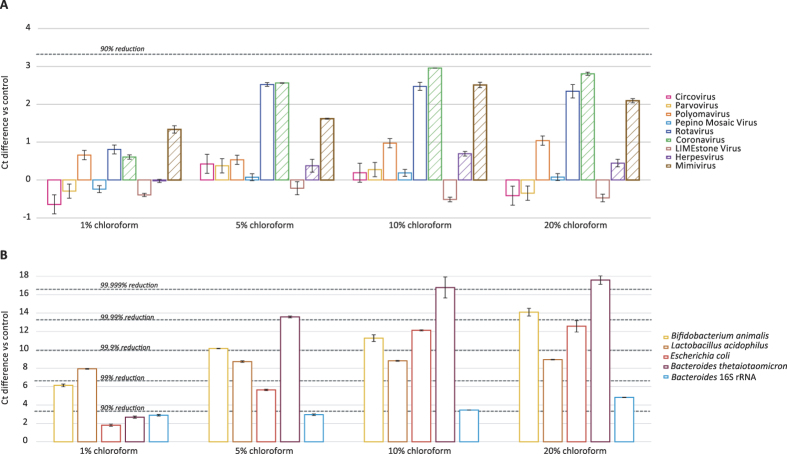
Ct differences vs control for chloroform treatment experiments performed on the mock-virome (A), bacterial mock-community and *Bacteroides* 16S rRNA (B). Enveloped viruses are depicted with a pattern. Standard deviations of the qPCR replicates are displayed.

**Figure 6:**
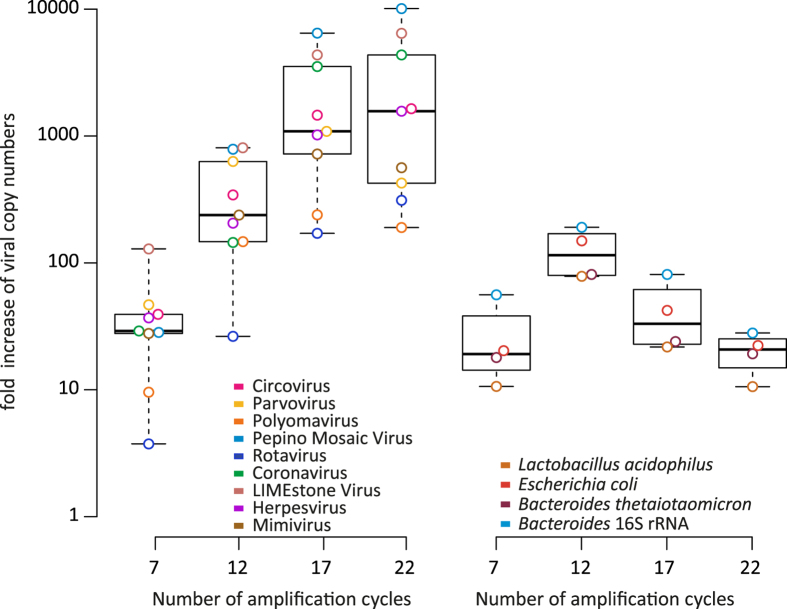
Fold increase vs control for random amplification experiments performed on the mock-virome, mock bacterial community and *Bacteroides* 16S rRNA. *Bifidobacterium animalis* is not shown since no amplification was observed.

Filtration is probably the most used procedure to separate viral from bacterial/host cells during virome studies. The most popular filters for viral metagenomics studies contain 0.45-μm or 0.22-μm pore sizes^17^. Our results showed that the majority (>99.5%) of bacteria in our mock-community were efficiently removed using filtration. Only the 0.8-μm PC filter had a relatively low filter efficiency (from 50% to 99.2%). Unfortunately, filters efficiently removing bacteria also removed more than 99% of mimivirus (Fig. 4A). Not unexpectedly, more than 90% of the herpesviruses were lost with the 0.22-μm filter, alongside unexpected losses of several smaller viruses. Free floating ribosomes (20–30 nm) were not efficiently removed by filtration regardless of the pore size. Our findings suggest that besides the pore size, also the filter material highly influences filter efficiency. This was prominently observed between 0.8-μm PC and PES filters. Polycarbonate filters have pores precisely cylindrical and narrowly distributed across a thin polycarbonate sheet, whereas the centrifugal PES filter is a 3D polymer network, resulting in much higher filtering efficiency for bacteria and mimivirus (Fig. 4). A limitation of our study is that no representatives of very small bacteria were included, however a great majority of small bacteria are obligate intracellular (e.g. Chlamydiales, Mycoplasmatales) and therefore removed together with their host during centrifugation and filtration.

Chloroform destabilises lipid membranes, disrupting the bacterial structural integrity after which bacterial nucleic acids become available for nucleases. According to our findings, chloroform concentration highly correlated with bacterial removal. However, enveloped viruses, especially coronavirus and mimivirus, also lost stability after the removal of the envelope. Interestingly, some non-enveloped viruses were also susceptible to chloroform, especially rotavirus and polyomavirus. It is also known that other non-enveloped phages belonging to the *Corticoviridae*, *Plasmaviridae* or *Inoviridae* are susceptible to chloroform^31^. The effect of chloroform seems highly virus-specific and therefore chloroform might not be a preferred approach for virome studies.

Random amplification of viral genetic material is crucial in viral metagenomics to obtain sufficient input material for sequencing. Many different amplification methods are available, some of which introduce substantial bias^19^,^32^. Of note, when using the WTA2 kit (ligating an oligonucleotide to either sides of the PCR products before amplification), we strongly recommend using the Nextera XT library preparation kit to avoid cluster-calling problems during Illumina sequencing. A limited disadvantage of using the Nextera XT library preparation kit is that the ends of viral genomes are underrepresented in the obtained reads due to the transposon technology use by this kit.

Kohl and colleagues showed that random amplification using a random primer with a universal 5′ end and a degenerate 3′ end was preferred to amplify viruses^20^. WTA2 utilises a similar approach and was modified to amplify both RNA and DNA. Although 17 amplification cycles are recommended by the manufacturer for transcriptome amplification, our findings indicate that all viruses could be amplified, independently of the number of cycles. However, the amplification efficiency differed between viruses, which could not be unambiguously explained by genome type (ssDNA, dsDNA, ssRNA, dsRNA), length or composition (linear, circular, segmented). In previous reports it was shown that multiple displacement amplification preferentially amplified (single-stranded) circular genomes, which was not observed in this study using WTA2^32^,^33^. Depending on the viral load in a sample, less than 17 amplification cycles can be sufficient, however, in our experience 17 amplification cycles produce reliable and reproducible results in various sample types with a wide range of virus concentrations. Nevertheless, in our protocol viral genome amplification introduced the most substantial bias, which was independent of the number of amplification cycles.

**Figure 7:**
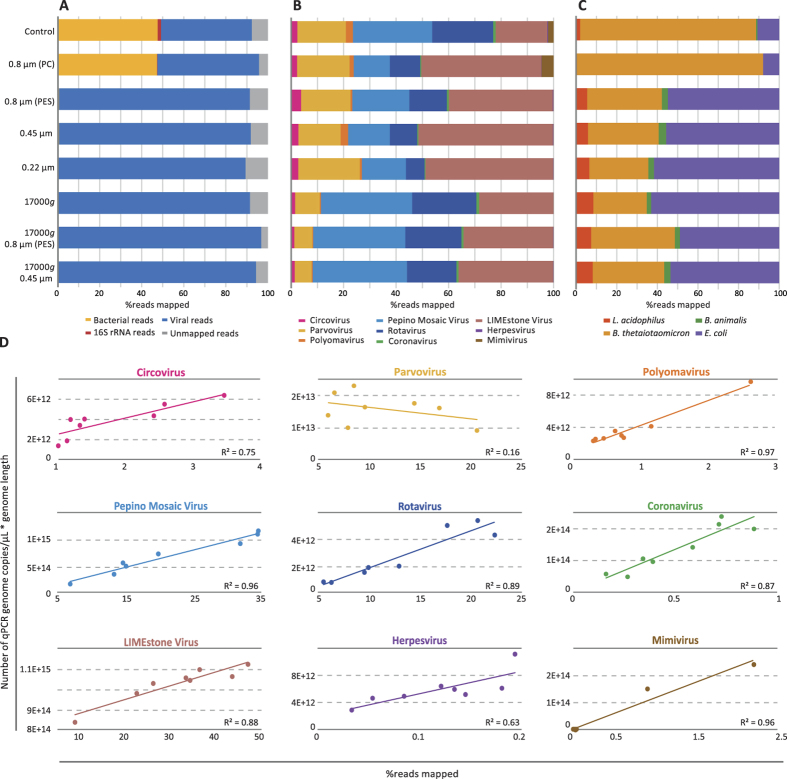
Percentage of NGS sequencing reads for bacterial, 16S rRNA, viral and unmapped reads for the conditions tested (**A**). Distribution of NGS sequencing reads for the mock-virome (**B**) and bacterial mock-community (**C**). Correlation between the percentage of mapped reads obtained per virus and the number of virus genome copies after amplification/μL*genome length as measured with qPCR (**D**). Normalization of the number of reads to the genome length allows for a proper comparison with qPCR results.

Independent of viral enrichment procedures, all viruses could be detected using the Illumina HiSeq2500™. The combination of centrifugation followed by filtration (0.8-μm PES) led to the highest number of viral reads (96.7%) and lowest percentage of bacterial and rRNA reads (0.19% and 0.003%; Fig. 7A). In other viral metagenomics studies a combination of more stringent centrifugation conditions and filtration, typically carried out using 0.45-μm or 0.22-μm pore sizes, have been applied^15^,^24^,^25^,^26^,^34^,^35^, which according to our results will lead to a dramatic reduction of larger viruses in a sample. Nevertheless, as with every viral enrichment protocol, including the one described in this study, the recovery of large viruses has to be balanced with the removal of bacterial/host genetic material, suggesting that bigger viruses (giant viruses, poxviruses, herpesviruses, etc.) might have been largely overlooked in past virome studies^28^.

Rosseel and colleagues^22^ showed that significant differences in sequence depth can be obtained when viral genomes are subjected to sequence independent amplification and Illumina sequencing, which could influence the relationship between qPCR results and read numbers. However, except for parvovirus, all viruses showed a high correlation between the number of viral genome copies measured by qPCR after amplification and the number of mapped NGS reads (R^2^=0.91). The aberrant behaviour of the parvovirus remains unexplained, but is unlikely to be an experimental error as good correlations between qPCR assays and NGS reads were obtained for the remaining viruses. Overall, this suggests that no additional strong bias are introduced after amplification. To extend this further, high reproducibility was shown for our preferred viral enrichment procedure (Fig. S1), which is crucial for comparison of viral abundancies from samples in large-scale studies.

The main aim of our study was to obtain a reproducible protocol which could be easily upscaled for high-throughput virome studies. CsCl ultracentrifugation poses difficulties with respect to reproducibility and automation, as discussed by Kleiner and colleagues^21^, and therefore was not investigated in this study. In addition, some viral families and genera are known to be unstable in CsCl, such as the *Guttaviridae, Orthomyxoviridae* and Nanovirus^31^. Furthermore a more recent study reported a strong loss of specific phages (ϕVPE25 and T7) tested when using a CsCl method^21^. For samples with high volume and low viral load (e.g. ocean waters, urine), an additional concentration step may be required. Approaches using FeCl_3_ or PEG precipitation have been shown very useful^36^,^37^,^38^. The quantitative effect of these procedures on each member of the viral community has not been studied. However, the use of a pre-filtration step with 0.2-μm, as often used for these methods, will lead to the removal of larger viruses (Fig. 4), which could be overcome by using a 0.8-μm filter instead.

In our method, only a single nucleic acids extraction method was tested. The QIAamp Viral RNA mini kit is a rapid method to extract viral nucleic acids, and has shown consistently good performance in previous virome studies^19^,^20^. Nevertheless, non-column based extraction methods might be considered to avoid silica contaminants^39^, but can be too time-consuming for large scale studies.

Although our optimisation was performed with mock communities containing high concentrations of viruses, NetoVIR has been successfully applied on a number of biological samples such as faeces (both of human and animal origin), clinical respiratory samples, serum samples and even homogenised insects. For all these samples a variety of known and unknown viruses could be identified.

To our knowledge this is the first modular VLP purification approach which provides virologists the information to adapt their procedures according to their particular needs. For virome studies in faecal samples we provide an overview and detailed explanation of NetoVIR (Fig. 1 and Protocol S1), which is suitable for large-scale studies and provided the best obtainable viral/bacterial ratio.

## Material and Methods

### Design of the mock-virome

The mock-virome consists of known concentrations (ranging from 10^7^ to 10^10^ genome copies per mL) of nine viruses (Table 1). These viruses were selected as representatives of a very wide range of physical virus characteristics, such as a distinct virus architecture (spherical, rod-shaped, head-tail structure), different virion sizes (ranging from 17 nm to 700 nm), presence or absence of an envelope, different genome lengths (ranging from 1.8 kb to 1180 kb) different genome types (dsDNA, dsRNA, ssDNA, ssRNA) and different genome compositions (linear, circular, segmented). The largest amount of the above diversity has been observed for eukaryotic viruses, explaining why only a single bacteriophage was included in our mock virome. Moreover, no (-)ssRNA virus was included as there is no reason to expect a different behaviour than any other ssRNA virus in any step of our procedure. We believe that the morphological and physicochemical properties of all know bacteriophages are covered by the nine viruses selected. Porcine circovirus 2, feline panleukopenia virus, feline infectious peritonitis virus and bovine herpesvirus were kindly provided by Sebastiaan Theuns and Hans Nauwynck (University of Ghent). The mimivirus was provided by Didier Raoult (Université de la Méditerranée). Inge Hanssen from Scientia Terrae VZW provided the pepino mosaic virus. The BK polyomavirus was provided by Robert Snoeck and Dimitrios Topalis (KU Leuven). The LIMEstone virus was obtained from Rob Lavigne (KU Leuven). Timo Vesikari (University of Tampere) provided the WC3 rotavirus. Except for the LIMEstone virus (arrived at 4 °C) all virus stocks were frozen upon arrival and were then thawn for q(RT)PCR quantification. Preparation of the mock communities was done with their original cell culture media, followed by aliquotation and freezing again at −80 °C before final use. For all the viruses, specific q(RT)-PCR assays and standards were either developed or retrieved from literature^40^,^41^,^42^ (Table S1).

### Design of the bacterial mock-community

The bacterial mock-community consists of known concentrations of four different bacteria representative for the majority of bacterial phyla in the gut (Table S2). *Lactobacillus acidophilus* (Firmicutes) and *Bifidobacterium animalis* are Gram-positive (Actinobacteria), whereas *Escherichia coli* (Proteobacteria) and *Bacteroides thetaiotaomicron* (Bacteroidetes) are Gram-negative. For each member in our mock-community, a qPCR assay targeting a unique and specific region was developed or retrieved from literature^43^. In addition, to quantify the number of 16S rRNA copies of the Bacteroides genus an additional qPCR assay was used (Table S3). The number of 16S copies for the other bacterial species was not determined because the structure of ribosomes is highly conserved among bacterial species, and were therefore not expected to behave differently in our experiments. All bacterial strains were acquired from the Belgian Co-ordinated Collection of Micro-organisms (BCCM) in their growing medium with the following LGM accession numbers: 13550 (*Lactobacillus acidophilus*), 2092 (*Escherichia coli*), 18906 (*Bifidobacterium animalis*) and 11262 (*Bacteroides thetaiotaomicron*). The bacterial mock-community was assembled from its original growing stocks and no buffers/medium were added and aliquots were made and preserved at −80 °C to ensure one freeze-thaw cycle.

### Real time (RT)-PCR assays

All qPCR assays for DNA viruses and bacteria were carried out using the TaqMan® Universal PCR Master Mix (Applied Biosystems) with the following conditions: 2 min at 50 °C, 10 min at 95 °C and 45 cycles of 15 sec at 95 °C and 1 min at 60 °C. RNA viruses and rRNA qRT-PCR were carried out with the One step qRT-PCR MasterMix Low Rox (Eurogentec) using the following conditions: 30 min at 48 °C followed by 10 min at 95 °C and 45 cycles of 15 sec at 95 °C and 1 min at 60 °C. qPCRs were performed on an ABI 7500 Real-Time PCR System (Applied Biosystems).

### Homogenisation

Mock-virome and bacterial mock-community were homogenised using a tissue homogeniser (MINILYS, Bertin technologies). A 200 μL stock of mock-virome or bacterial mock-community were subjected to different homogenisation speeds (3000 rpm or 5000 rpm) with or without the presence of ceramic beads (Ø0.1 mm (CK01–2 ml, Precellys) or Ø2.8 mm (CK28–2 ml, Precellys)). All samples were homogenised for 1 min. After homogenisation, samples were treated for 2 hours at 37 °C with a cocktail of 1 μl microccocal nuclease (NEB) and 2 μl of benzonase (Millipore) and 7 μl of homemade buffer (1M Tris, 100 mM CaCl_2_ and 30 mM MgCl_2_, pH 8) and extracted with the QIAamp Viral RNA Mini Kit (Qiagen) without carrier RNA.

### Centrifugation

Samples were centrifuged using a bench top centrifuge (Heraeus pico 17, Thermoscientific). Two-hundred  μl of mock-virome or bacterial mock-community was centrifuged at 100 *g* or 17000 *g* for 3 min or 30 min. Subsequently, samples underwent nuclease treatment, nucleic acids extraction and qPCR assays as described above.

### Filtration

Aliquots of 1000 μl containing mock-virome or bacterial mock-community were filtered through a 0.8-μm centrifugal (PES) filter (VK01P042, Sartorius), a 0.8-μm polycarbonate (PC) filter (ATTP14250, Millipore), a 0.45-μm centrifugal filter (UFC40HV00, Millipore) or a 0.22-μm centrifugal filter (UFC40GV00, Millipore). The 0.8-μm PC filter was pre-wetted with nuclease-free water prior to filtration to enhance performance. After filtration, samples underwent a nuclease treatment, nucleic acids extraction and qPCR assays as described above.

### Chloroform treatment

Mock-virome or bacterial mock-community (150 μl) were incubated with 1%, 5%, 10% and 20% (%v/v) of chloroform and incubated for 60 min in a ferris wheel at 10 rpm (model L23, Labinco BV). After the chloroform treatment, samples underwent a nuclease treatment, nucleic acids extraction and qPCR assays as described above.

### Random amplification

Random amplification of nucleic acids was performed using the Whole Transcriptome Amplification Kit 2 (WTA2, Sigma Aldrich) according to manufacturer’s instructions with the exception of the initial denaturation step which was performed at 95 °C instead of 70 °C in order to also denature double-stranded DNA or RNA to make it available for the amplification. In addition, the number of amplification cycles was varied between 7, 12, 17 and 22. WTA2 products were purified with the QIAquick PCR Purification Kit (Qiagen) according to the manufacturer’s instructions and amplification efficiency was determined for every virus and bacteria using qPCR assays as described above.

### Illumina sequencing

Mock-virome and bacterial mock-community were pooled in 1:1 ratio to a total of 1.5 mL and homogenised for 1 min at 3000 rpm with a MINILYS homogeniser. Aliquots of the pooled sample were then centrifuged for 3 min at 17000 *g*, and/or filtered through 0.8 (PES/PC) μm or 0.45-μm/0.22-μm filter. Samples underwent a nuclease treatment, nucleic acids, extraction and random amplification as previously described. qPCR assays were performed for the mock-virome and the bacterial mock communities’ amplified WTA product. An NGS library preparation was performed using the Nextera XT DNA Library Preparation kit (Illumina) according to the manufacter’s instructions except that 1) the tagmentation time was decreased to 4 min, 2) input DNA was increased to 1.2 ng/μl and 3) reagents’ quantities were halved to increase the average size of the DNA fragments. Fragment sizes were determined using a High Sensitivity DNA Kit (Agilent) and run in a Bioanalyzer 2100 (Agilent). Libraries were quantified with the KAPA Library Quantification kit (Kapa Biosystems) and sequencing was performed on a HiSeq™ 2500 platform (Illumina) for 2 × 150 cycles. Each sample was attributed a total of 10 million paired end reads.

The obtained sequence reads were subjected to a bioinformatics pipeline comprising deduplication (FastUniq)^44^, quality and adapter trimming (Trimmomatic)^45^, and subsequently mapped with Burrows-Wheeler Aligner (BWA)^46^ against reference genomes of all viruses, bacteria and 16S rRNA present in the mock-virome and bacterial mix. The number of mapped reads for each genome was obtained with Samtools^47^ and the percentage of mapped reads was calculated as the number of mapped reads divided by the total number of reads after trimming. For every virus, the correlation between the qPCR results and the percentage of mapped reads was determined based on all viral enrichment conditions. Normalisation of the number of reads to the genome length allows for a proper comparison with qPCR results.

### Method validation: reproducibility

Mock-virome and bacterial mock-community were pooled in 1:1 ratio, and split into two aliquots. From both aliquots a part was kept for qPCR before treatment and underwent a nuclease treatment and nucleic acids extraction. The rest of the mock-virome and bacterial mock-community were treated as follows: homogenisation was performed for 1 min (3000 rpm), followed by centrifugation for 3 min at 17000 *g*, then filtration with the 0.8-μm PES. Samples underwent a nuclease treatment, nucleic acids extraction and a random amplification was performed and qPCR assays were done as described above.

## Additional Information

**How to cite this article**: Conceição-Neto, N. *et al.* Modular approach to customise sample preparation procedures for viral metagenomics: a reproducible protocol for virome analysis. *Sci. Rep.* **5**, 16532; doi: 10.1038/srep16532 (2015).

## Supplementary Material

Supplementary Information

## Figures and Tables

**Table 1:** List of nine selected viruses present in the mock-virome together with their classification, host species and physical characteristics.

Virus/phage strain	Virus family	Host	Baltimore classification	Shape	Genome composition	Genome size (kb)	Virion size (nm)	Enveloped?	Bouyant Density in CsCl (g cm^−2^)	Number of viral genome copies/mL
Porcine circovirus 2 (10-10)	*Circoviridae*	Pigs	Group II: ssDNA	Spherical	Circular	1.8	≈17	NO	1.37	1.62 × 10^9^
Feline panleukopenia virus	*Parvoviridae*	Cats	Group II: ssDNA	Spherical	Linear	5	≈20	NO	1.40	2.09 × 10^10^
Polyomavirus (BKV)	*Polyomaviridae*	Humans	Group I: dsDNA	Spherical	Circular	5	≈50	NO	1.34	1.34 × 10^9^
Pepino Mosaic virus (CH2)	*Alphaflexiviridae*	Tomato plant	Group IV: ssRNA (+)	Rod	Linear	6	≈470-800 × 12–13	NO	1.31	1.34 × 10^9^
Rotavirus A (WC3)	*Reoviridae*	Cows	Group III: dsRNA	Spherical	Segmented	19	≈100	NO	1.36–1.39	8.57 × 10^8^
Feline infectious peritonitis virus (serotype 1683)	*Coronaviridae*	Cats	Group IV: ssRNA (+)	Spherical	Linear	30	≈100	YES	1.23–1.24	8.76 × 10^8^
Bovine herpesvirus 1 (Cooper strain)	*Herpesvirus*	Cattle	Group I: dsDNA	Spherical	Linear	135	≈200	YES	1.23	4.16 × 10^7^
LIMEstone (vB_DsoM_LIMEstone1)	*Myoviridae*	Bacterium Dickeya solani (potato pathogen)	Group I: dsDNA	Head-tail structure	Linear	152	≈91 (head), 114 × 17 (tail)	NO	1.50	1.69 × 10^8^
Acanthamoeba polyphaga mimivirus	*Mimiviridae*	Acanthamoeba polyphaga (Amoeba)	Group I: dsDNA	Spherical	Linear	1181	≈500-700	YES (Internal)	1.36	1.47 × 10^8^
