# Supplementary material for: Modular approach to customise sample preparation procedures for viral metagenomics: a reproducible protocol for virome analysis
Source: Sci Rep. 2015 Nov 12;5:16532. doi: 10.1038/srep16532 (PMC4642273; doi:10.1038/srep16532)
Supplement: Supplementary Information [file srep16532-s1.pdf]

Table S1 – Primers, probes and standards used to perform qPCR on nine viruses of the mock-virome.

		**Sequence (5' – 3')**
Porcine circovirus 2 (10-10)	**Fw primer**	CCGTTGGAATGGTACTCCTC
	**Probe**	FAM-CTGCTGTCCCAGCTGTAGAAGCTCTYT-TAMRA
	**Rv primer**	CCAAGGAAGTAATCCTCCGA
	**Standard**	AATCAGACCCCGTTGGAATGGTACTCCTCAACTGCTGTCCCAGCTGTAGAAGCTCTYTATCGGAGGATTACTTCCTTGGTATTTTGGA
Feline panleukopenia virus	**Fw primer**	CATGGAAACCAACCATACCA
	**Probe**	FAM-CTGGAACTAGTGGCACACCAACAAATG-TAMRA
	**Rv primer**	TGAACATCATCTGGATCTGTACC
	**Standard**	TTTTTATCCATGGAAACCAACYATACCAAGTCCATGGAGATATTATTTTCAATGGGATAGAACATTAATACCATCTCATACTGGAACTAGTGGCACACCAACAAATRTATATCATGGYACAGATCCAGATGATGTTCAATTTTATA
Polyomavirus (BKV)	**Fw primer**	GGGTGCTGCTCTAGCACTTT
	**Probe**	FAM-TCCTGTGGCAGCAGCAGCCT-TAMRA
	**Rv primer**	TAGCAAGGGATGCAATTTGA
	**Standard**	GGGTGCTGCTCTAGCACTTTTGGGGGACCTAGTTGCCAGTGTATCTGAGGCTGCTGCTGCCACAGGATTTTCAGTGGCTGAAATTGCTGCTGGGGAGGCTGCTGCTGCTATAGAAGTTCAAATTGCATCCCTTGCTA
Pepino Mosaic virus (CH2)	**Fw primer**	TGGGTTTAGCAGCCAATGAGA
	**Probe**	FAM-CGGACCTGCCATGTGGGACCTC-TAMRA
	**Rv primer**	AACTTTGCACATCAGCATAAGCA
	**Standard**	TTACTGCCATGGGTTTAGCAGCCAATGAGACCGGACCTGCCATGTGGGACCTCGCTCGTGCTTATGCTGATGTGCAAAGTTCAAAATCTG
Bovine rotavirus A (WC3)	**Fw primer**	GGCTTTTAATGCTTTTCAGTGGT
	**Probe**	FAM- CTCAAGATGGAGTCTACTCAGCAGATG-TAMRA
	**Rv primer**	GCAGCAACGACTGCGGCTTC
	**Standard**	GGCTTTTAATGCTTTTCAGTGGTTGATGCTCAAGATGGAGTCTACACAGCAGATGGCTAGTTCGATTATTAACACTTCTTTTGAAGCCGCAGTCGTTGCTGC
Feline infectious peritonitis (serotype 1683)	**Fw primer**	GATTTGATTTGGCAATGCTAGATTT
	**Probe**	FAM-TCCATTGTTGGCTCGTCATAGCGGA-TAMRA
	**Rv primer**	AACAATCACTAGATCCAGACGTTAGCT
	**Standard**	GAAGTTTAGATTTGATTTGGCAATGCTAGATTTAGTAATTTAGAGAAGTTTAAAGATCCGCTATGACGAGCCAACAATGGAAGAGCTAACGTCTGGATCTAGTGATTGTTTAAAATGT
Bovine herpesvirus 1 (Cooper strain)	**Fw primer**	TGTGGACCTAAACCTCACGGT
	**Probe**	FAM-AGGACCGCGAGTTCTTGCCGC-TAMRA
	**Rv primer**	GTAGTCGAGCAGACCCGTGTC
	**Standard**	CAGCACCTTTGTGGACCTAAACCTCACGGTTCTGGAGGACCGCGAGTTCTTGCCGCTAGAAGTGTACACGCGCGCCGAGCTCGCCGACACGGGTCTGCTCGACTACAGCGAGATA
LIMEstone (vB_DsoM_LIMEstone1)	**Fw primer**	TGCTCTGGTTCAGTCTGGTC
	**Probe**	FAM-CCATCTTCGTCAATCTCAATGTCGGA-TAMRA
	**Rv primer**	GCGCACTGATTTGTAGAGGA
	**Standard**	TGCTCTGGTTCAGTCTGGTCGCATCTACGAGTTCATGGCATACTCCGACATTGAGATTGACGAAGATGGTGACATCATCCTCTACAAATCAGTGCGC
Acanthamoeba polyphaga mimivirus	**Fw primer**	GATGACCAGCTTGTTTCCCT
	**Probe**	FAM-TCATCAAGAGGTCATCCAAGATCGACA-TAMRA
	**Rv primer**	AAGTCTGTCGATGAAGCTACCA
	**Standard**	TTGGTGCGAGATGACCAGCTTGTTTCCCTTAATAGAGAGTTGGAGTTTTTCAAGCTCTTTGTCGATCTTGGATGACCTCTTGATGATCTTACCGATAAGCTTACTGGTAGCTTCATCGACAGACTTATTGGTAGT

Table S2 – List of the four selected bacteria present in the bacterial mock community, enclosing their characteristics.

**Bacterial strain**	**Phylum**	**Gram stain**	**Aerobic/Anaerobic**	**Dimensions (nm)**	**Genome size (Mb)**	**Number of bacterial genome copies/mL**
Bacteroides thetaiotaomicron	*Bacteroidetes*	Negative	Anaerobic	700-1000 x 1000-2000	6.26	$4.07x10^{7}$
Escherichia coli	*Proteobacteria*	Negative	Anaerobic	250-1000 x 2000	4.6	$2.51x10^{6}$
Lactobacillus acidophilus	*Firmicutes*	Positive	Aerobic	500-900 x 1500-9000	1.85	$1.77x10^{6}$
Bifidobacterium animalis	*Actinobacteria*	Positive	Anaerobic	600-900 x 1500-6000	1.9	$3.28x10^{6}$

Table S3 – Primers, probes and standards used to perform qPCR on the four bacteria.

		**Sequence (5' – 3')**	**Target gene**
Lactobacillus acidophilus	**Fw primer**	AAGTTGATGCAGATGTTCAAGTC	rpoA gene
	**Probe**	FAM – CAAGTGTCCACCTTCAGCAATGGTACA - TAMRA	
	**Rv primer**	TACGGCGAGTTCCATGTG	
	**Standard**	AAGTTGATGCAGATGTTCAAGTCTTAAATCCAGATCAATATATTTGTACCAT<br>TGCTGAAGGTGGACACTTGCACATGGAACTCGCCGTA	
Bifidobacterium animalis	**Fw primer**	AACAAGATGGCACCTCCATT	RecA gene
	**Probe**	FAM –TGCGAGATGCCCTGCCCATA - TAMRA	
	**Rv primer**	ATGTCGAGAATCGACCCTTC	
	**Standard**	AACAAGATGGCACCTCCATTCAAGACCGCCGAGTTCGACATGCTCTATGGG<br>CAGGGCATCTCGCAGGAAGGGTCGATTCTCGACAT	
Escherichia coli	**Fw primer**	GGCTTCCTTGAGACTCCGTA	rpoB gene
	**Probe**	FAM –CGTCAGTTACAACACCGTCGGTCA- TAMRA	
	**Rv primer**	GGCGATAACGTAGTTGCCTT	
	**Standard**	GGCTTCCTTGAGACTCCGTATCGTAAAGTGACCGACGGTGTTGTAACTGAC<br>GAAATTCACTATCTGTCTGCTATCGAAGAAGGCAACTACGTTATCGCC	
Bacteroides thetaiotaomicron	**Fw primer**	AGCAGATTCTTCTGCCCTGT	bexA gene
	**Probe**	FAM –CGCGATGCATGGAACTGATTCC- TAMRA	
	**Rv primer**	AAAGTGATTTCCGCTACGCT	
	**Standard**	AGCAGATTCTTCTGCCCTGTTTTTCAACGCGATGCATGGAACTGATTCCAAA<br>GTAAGCATCCAGAACAGCGTAGCGGAAATCACTTT	
Bacteroides	**Fw primer**	GAGAGGAAGGTCCCCCAC	16S rRNA
	**Probe**	FAM – CCATTGACCAATATTCCTCACTGCTGCCT- TAMRA	
	**Rv primer**	CGCTACTTGGCTGGTTCAG	
	**Standard**	GAGAGGAAGGTCCCCCACATTGGAACTGAGACACGGTCCAAACTCCTACG<br>GGAGGCAGCAGTGAGGAATATTGGTCAATGGACGAGAGTCTGAACCAGCC<br>AAGTAGCG	

Table S4 – Reads obtained from the Illumina Hiseq 2500 run.

	**Control**	**0.8 PC**	**0.8 PES**	**0.45**	**0.22**	**17000*g***	**17000*g*+0.8 PES**	**17000*g*+0.45**
**Circovirus**	114327 (1.01%)	105066 (1.09%)	467836 (3.47%)	288199 (2.56%)	370639 (2.42%)	162676 (1.39%)	160240 (1.18%)	174241 (1.32%)
**Parvovirus**	902175 (8.00%)	929352 (9.68%)	2304080 (17.07%)	1645670 (14.64%)	3189729 (20.81%)	1008512 (8.59%)	904778 (6.66%)	802191 (6.06%)
**Polyomavirus**	130761 (1.16%)	72391 (0.75%)	88285 (0.65%)	294442 (2.62%)	111870 (0.73%)	47493 (0.40%)	43676 (0.32%)	45715 (0.35%)
**Pepino mosaic virus**	1468324 (13.02%)	639869 (6.67%)	2660872 (19.72%)	1623254 (14.44%)	2279153 (14.87%)	3710870 (31.63%)	4610257 (33.91%)	4479201 (33.85%)
**Rotavirus**	1132196 (10.04%)	535543 (5.58%)	1751352 (12.98%)	1078350 (9.59%)	971060 (6.34%)	2619165 (22.32%)	2812629 (20.69%)	2350602 (17.76%)
**Coronavirus**	44067 (0.39%)	15438 (0.16%)	79082 (0.59%)	38486 (0.34%)	40884 (0.27%)	104041 (0.89%)	98880 (0.73%)	94547 (0.71%)
**LIMEstone virus**	960756 (8.52%)	2125479 (22.15%)	4860630 (36.02%)	5252016 (46.71%)	6612059 (43.15%)	3024671 (25.78%)	4484817 (32.99%)	4487085 (33.91%)
**Herpesvirus**	11958 (0.11%)	4007 (0.04%)	32653 (0.24%)	25407 (0.23%)	23196 (0.15%)	7903 (0.07%)	24651 (0.18%)	22206 (0.17%)
**Mimivirus**	103091 (0.91%)	211129 (2.20%)	3711 (0.03%)	2809 (0.02%)	5240 (0.03%)	3133 (0.03%)	1594 (0.01%)	2386 (0.02%)
***Bacteroides* rRNA**	179167 (1.59%)	18923 (0.20%)	1694 (0.01%)	966 (0.01%)	559 (0.004%)	1026 (0.01%)	455 (0.003%)	463 (0.003%)
Bacteroides	4655437 (41.27%)	4172023 (43.47%)	34282 (0.25%)	27823 (0.25%)	26408 (0.17%)	12152 (0.10%)	10828 (0.08%)	11988 (0.09%)
Bifidobacterium	38488 (0.34%)	2474 (0.03%)	2748 (0.02%)	2935 (0.03%)	2508 (0.02%)	988 (0.01%)	670 (0.005%)	1060 (0.01%)
Lactobacillus	107010 (0.95%)	5289 (0.06%)	5170 (0.04%)	4693 (0.04%)	5894 (0.04%)	3957 (0.03%)	1958 (0.01%)	2806 (0.02%)
E.coli	572592 (5.08%)	357029 (3.72%)	51191 (0.38%)	44481 (0.40%)	55943 (0.37%)	29121 (0.25%)	12829 (0.09%)	18254 (0.14%)
**unmapped**	859446 (7.62%)	403677 (4.21%)	1152082 (8.54%)	914920 (8.14%)	1629299 (10.63%)	998115 (8.51%)	426666 (3.14%)	740146 (5.59%)
**TOTAL**	11279795	9597689	13495668	11244451	15324441	11733823	13594928	13232891

Table S5 – Standard deviation obtained in the reproducibility experiment for viruses and bacteria

	**Standard deviation before NetoVIR (%)**	**Standard deviation after NetoVIR (%)**
**Circovirus**	13.00	23.81
**Parvovirus**	9.04	15.08
**Polyomavirus**	19.30	13.96
**Pepino mosaic virus**	5.66	4.96
**Rotavirus**	9.71	3.26
**Coronavirus**	8.09	3.11
**LIMEstone virus**	2.68	8.66
**Herpesvirus**	6.59	4.55
**Mimivirus**	12.87	38.41
***Bacteroides* 16S rRNA**	9.53	18.37
Bacteroides	1.14	Below detection limit
Bifidobacterium	6.65	Below detection limit
Lactobacillus	18.39	Below detection limit
E.coli	29.26	21.04

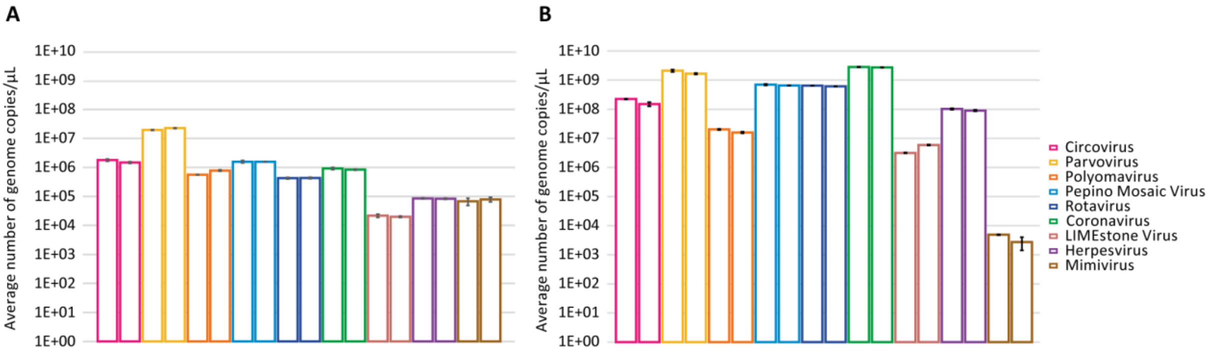

Figure S1 – Average number of genome copies of viruses per µL before (A) and after (B) performance of the optimised protocol with two replicates.

Protocol S1 – NetoVIR (Novel enrichment technique of VIRomes)

MATERIAL

Equipment

- MINILYS homogeniser (Bertin technologies)
- Benchtop centrifuge
- Microcentrifuge
- Thermocycler
- Magnetic rack (for 96 well plates)
- Heating block
- Real-time PCR instrument
- Qubit Fluorometer
- 2 mL empty tubes and caps with O-ring for MINILYS (Cat No: KT03961-1-405.2) (VWR)
- Filter with PES membrane of 0.8-µm (Sartorius Vivaclear Mini VK01P042)
- Filtered tips
- Empty 1.5 mL and 2 mL tubes
- Qubit® Assay Tubes, (ThermoFisher Scientific, Catalog number: Q32856)
- 96-well plates

Reagents

- Sterile PBS
- Tris, $CaCl_2$, $MgCl_2$ and EDTA
- 100% ethanol
- Benzonase Nuclease, Purity > 90% (Millipore Cat. No: 70746-3)
- Micrococcal nuclease (New England Biolabs Cat. No: M0247S)
- Agencourt AMPure XP beads 5 mL (Beckman Coulter Cat. No: A63880)
- 10 mM Tris-HCl, pH 8.0

Kits

- QIAamp® Viral RNA Mini kit (Qiagen, Cat. No (50 preps): 52904 or Cat. No (250 preps): 52906)
- WTA2 - Complete Whole Transcriptome Amplification Kit (Sigma Aldrich, WTA2-10RXN or WTA2-50RXN for 50 and 250 reactions, respectively).
- Nextera XT DNA Library preparation (Illumina, Cat. No: FC-131-1024 or FC-131-1096)
- Nextera XT Index Kit (24 or 96 indices) (Illumina, Cat. No: FC-131-2001 and FC-131-2004, other options available: check Illumina website)
- Universal KAPA Library quantification kit (KAPA Biosystems, Cat. No: KK4824)
- Qubit™ dsDNA HS Assay Kits (Life Technologies, Cat. No (100 assays): Q32851 or (500 assays): Q32854)
- MSB® SPIN PCRAPACE (Stratec Biological, Cat. No (50 preps): 1020220200, (250 preps): 1020220300 or (500 preps): 1020220400)

PROCEDURE

CRITICAL STEP: During the whole procedure it is crucial to keep all working spaces sterile.

Faecal sample dilution ♦ TIMING: 5 min per sample

1| Weigh the faecal material (≈50 mg) and add 500 µL of sterile PBS to attain a 10% (m/v) suspension.

Sample homogenisation ♦ TIMING: 1 min per sample

2| Set the MINILYS homogeniser for 3000 rpm (speed 1) for 60 seconds using 2 mL tubes with O-ring caps.

CRITICAL STEP: Tubes should not have any type of beads, this results in massive loss of viruses. Without homogenisation filters are more likely to clog.

Centrifugation ♦ TIMING: 3 min

3| Centrifuge at 17000*g* for 3 min. Retrieve at least 150 µL of the supernatant for the next step.

Filtration ♦ TIMING: 1 min per sample

4| Filter the supernatant obtained in a 0.8 µm (PES) filter at 17000*g* for 1 min.

TROUBLESHOOTING: In case of clogging of the filter membrane consider further diluting the sample or replace the spin membrane with a new one and transfer the remaining supernatant. Alternatively, a pre-filtration step with a larger pore size of 10 µm could be performed.

Nuclease treatment ♦ TIMING: 2h

5| Set a heating block for 37˚C.

6| Add 7 µL of 20x homemade buffer (1M Tris, 100 mM $CaCl_2$ and 30 mM $MgCL_2$, pH=8) to 130 µL of sample filtrate.

7| Add 2 µL of benzonase and 1µL of micrococcal nuclease.

8| Mix by gently inverting the tubes 3 times.

9| Incubate for 2h at 37˚C

10| Stop the reaction by adding 7 µL of 10 nM EDTA and freeze samples at –80°C for 24h **or preferably** proceed for extraction.

▪ PAUSE POINT

Extraction ♦ TIMING: ≈25 min for 1 sample. 1h30min for 24 samples.

Extraction is performed using the QIAamp® Viral RNA Mini kit from Qiagen, without carrier RNA. Change gloves between steps and use aerosol-barrier tips. To avoid contamination do not place tubes

immediately next to each other in the rack. Please note that even though it is an RNA extraction kit, DNA will also be extracted.

11| Mix in an RNAse-free 2 mL tube the following:

Component	Volume (µL)	Purpose
AVL buffer	560	Lysis
Sample after nuclease treatment	140	-

CRITICAL STEP: Do **not** add carrier RNA to the AVL buffer.

12| Mix the mixture by pulse vortexing.

13| Incubate at room temperature for 10 min.

14| Change gloves.

15| Briefly centrifuge the mixture with a microcentrifuge.

16| Add 560 µL of absolute ethanol to the sample mixture.

CRITICAL STEP: Mix very well by pulse-vortex.

17| Briefly centrifuge the mixture with a microcentrifuge.

18| Add 630 µL of the sample mixture to the spin column.

CRITICAL STEP: Do not touch the column rim with the pipet.

19| Centrifuge at 6000*g* for 1 min, change the collection tube and repeat steps 18 and 19.

20| Add to the spin column the following:

CRITICAL STEP: Prepare the AW1 buffer according to the guidelines of the supplier, as it is originally as a concentrate.

Component	Volume (µL)	Purpose
AW1 buffer	500	DNA and RNA purification

21| Centrifuge at 6000*g* for 1 min, change the collection tube.

22| Add to the spin column the following:

CRITICAL STEP: Prepare the AW2 buffer, as it is originally as a concentrate.

Component	Volume (µL)	Purpose
AW2 buffer	500	DNA and RNA purification

23| Centrifuge at 20 000*g* for 3 min, change the collection tube.

24| Centrifuge at 20 000*g* for 1 min. Place the spin column in an RNAse-free 1.5 mL tube.

25| Add 60 µL of AVE (elution buffer) to the spin column and incubate for 1 minute.

CRITICAL STEP: Pipet the AVE buffer directly onto the filter membrane without touching it with the pipet tip.

26| Centrifuge at 6000*g* for 1 min.

27| Store viral RNA and DNA at –80°C.

▪ **PAUSE POINT**

Amplification ♦ TIMING: 3h30

Amplification is performed using a modified WTA2 (Complete Whole Transcriptome Amplification Kit) protocol from Sigma Aldrich.

CRITICAL STEP: The number of cycles chosen depends on the input material. Typically we recommend 17 cycles, however for samples with expected low viral DNA and RNA content, an increase to 22 cycles should be considered. Similarly for samples with a high level of input material, 12 cycles can be used.

Component	Volume (µL)
Library Synthesis Solution	0.5
Extracted viral sample	2.82

29| Mix by pipetting and incubate in a thermocycler programmed for 95°C for 2 minutes then cool down to 18°C.

30| To the cooled-primed DNA and RNA (18°C) sample, immediately add the following:

Component	Volume (µL)
Library Synthesis Buffer	0.5
RNAse free water	0.78
Library Synthesis Enzyme	0.4

31| Mix and incubate in a thermocycler programmed for:

18°C for 10 minutes

25°C for 10 minutes

37°C for 30 minutes

42°C for 10 minutes

70°C for 20 minutes

4°C

32| To the DNA library add the following mix to each sample:

Component	Volume (µL)
Amplification mix	7.5
RNAse free water	60.2
WTA dNTP mix	1.5
Amplification enzyme	0.75
DNA library	5

33| Mix by pipetting and incubate in a thermocycler programmed for:

94°C for 2 minutes.

94°C for 30 seconds|

70°C for 5 minutes |17 cycles

34| Purify the PCR product using any standard PCR purification kit. We use the MSB® SPIN PCRAPACE from Stratec as follows:

35| Add 500 µL of binding buffer to the 75 µL of WTA2 amplification product.

CRITICAL STEP: Binding buffer is a concentrate, 99.7% isopropanol needs to be added accordingly.

36| Place the spin filter in a 2 mL receiver tube and add the 575 µL of mixture.

37| Centrifuge at 11000*g* for 3 min.

38| Place the spin filter in a 1.5 mL receiver tube. Elute in 50 µL of elution buffer.

39| Incubate for 1 min.

40| Centrifuge at 11000*g* for 1 min.

41| Store amplified DNA at –80°C.

▪ PAUSE POINT

WTA2 amplification product concentration measurement using Qubit ♦ TIMING: ≈5-10 min

42| Measure the concentration of each WTA2 purified product by Qubit.

Concentration is measured using the Qubit dsDNA HS assay kit, which provides 2 standards. Tubes should be purchased separate.

43| Set up the required number of 0.5 mL Qubit tubes for the 2 standards and samples (*n* samples +2).

44| Prepare a working solution by adding per sample 199 µL of Qubit dsDNA HS Buffer and 1 µL of Qubit dsDNA HS Reagent. We recommend to prepare 10% extra of working solution to assure enough volume.

45| Load 190 µL of working solution in the standard tubes and 199 µL in the tubes for samples.

46| Add 10 µL of standard to the respective tubes and 1 µL of sample in each sample tube.

47| Mix by pulse-vortexing and incubate for 2 minutes at room temperature.

48| On the Home Screen of the Qubit Fluorometer, press dsDNA High Sensitivity as the assay type and run a new calibration.

49| Read standard #1 and standard #2.

50| Read the samples.

51| Calculate concentration of samples

$$Concentration\ of\ your\ sample\ in\ ng/ml = Qubit\ value\ x\ (\frac{200}{\mu L\ of\ sample})$$

Library preparation ♦ TIMING: ≈1h

Library preparation is performed using an adjusted protocol for the Nextera XT DNA sample preparation kit from Illumina.

CRITICAL STEP: Perform the following reactions on ice.

CRITICAL STEP: Make a dilution from the WTA2 purified product according to the Qubit measurement to 1.2 ng/µl to be used as input material.

CRITICAL STEP: The number of cycles chosen (15) and the extension time depends on the input material and the optimal fragment size. In this protocol, the tagmentation time was decreased from 5 to 4 min (resulting in longer fragments) and the PCR extension was increased to 45 sec. Consider testing other combinations of conditions for optimal results with your specific samples. Additional guidance can be found in the library preparation section of the study of Picelli *et al* (Full-length RNA-seq from single cells using Smart-seq2, 2013).

52| Mix the following in 200 µl PCR tubes in order to achieve DNA tagmentation:

Component	Volume (µL)
Tagment Buffer	5
Amplicon tagment mix	2.5
Amplified DNA (1.2 ng/µl)	2.5

53| Centrifuge at 280*g* for 1 min (or using a microcentrifuge for 1 min)

54| Incubate in a thermocycler for 4 min at 55°C, followed by cooling to 10°C and proceed immediately to next step.

55| Stop tagmentation by adding the following to the 10µl of tagmented DNA :

Component	Volume (µL)
Neutralize Tagment Buffer	2.5

56| Centrifuge at 280*g* for 1 min (or using a microcentrifuge for 1 min)

57| To the tagmented DNA (12.5 µl) add the following:

Component	Volume (µL)
Primer 1 (i7, N70X)	2.5
Primer 2 (i5, S50X)	2.5
Nextera PCR master mix	7.5

CRITICAL STEP: Nextera primers are not provided together with the library preparation kit and need to be purchased separate. A careful choice of primers is advised to assure unique combinations of indices per sample.

58| Centrifuge at 280*g* for 1 min (or using a microcentrifuge for 1 min)

59| Incubate in a thermocycler programmed for:

72°C for 3 minutes

95°C for 30s

95°C for 10 s|

55°C for 30 s|

72°C for 45 s |15 cycles

4°C

60| Store amplified DNA at 4°C for **maximum** 24h or proceed to PCR clean-up.

▪ PAUSE POINT

PCR purification using AMPure beads♦ TIMING: 45 min

61| Prepare fresh 80% ethanol and vortex AMPure beads for a few seconds.

62| Mix the following by pipetting until an homogeneous solution is obtained

Component	Volume (µL)
PCR product	25
AMPure beads	15

63| Incubate at room temperature for 5 min.

59| Place the plate in the magnetic stand for 2 min.

60| VERY carefully remove and discard supernatants.

61| Keeping the plate on the magnetic stand, add 200 µl of 80% ethanol to each sample.

62| Incubate the plate/tube at room temperature for ≥ 30s.

63| Carefully remove and discard the ethanol. **Repeat this step once.**

64| Try to remove all residual ethanol by pipetting. Incubate at room temperature for about 3 min without drying the beads.

CRITICAL STEP: Watch the beads and if you observe cracks, proceed for next step immediately. The time can be highly variable depending on the temperature and humidity inside the lab.

65| Thoroughly resuspend the beads in 26.25 µl of 10 mM Tris-HCl (pH 8.0) by pipetting them up and down ≈10 times.

66| Incubate the plate at room temperature for 2 min to elute DNA from the beads.

67| Place the plate on the magnetic stand for 2 min.

68| Carefully transfer 25 µl of the supernatant to new RNAse free tubes.

69| Store amplified DNA at –80°C.

▪ PAUSE POINT

DNA library concentration measurement using Qubit ♦ TIMING: ≈5-10 min

70| Measure the concentration of each library by Qubit as described from step **42|** to **51|**

DNA library size distribution and quality check using the Bioanalyzer ♦ TIMING: ≈1h

71| Check the size distribution on an Agilent high-sensitivity chip (Bioanalyzer). Each chip can load 11 samples.

72| Remove all reagents from the fridge (4°C) and allow them to reach room temperature for around 30 minutes before use. Keep dye and prepared gels from light.

73| Set up the chip priming station's lever to the lowest position. Adjust the IKA vortex mixer to 2400 rpm.

74| Start the software, select assays: High Sensitivity DNA assay. Name your sample wells on the software.

75| Prepare the gel-dye mix by adding 15 µL of dye (blue-capped tube) to the red-capped tube.

76| Vortex the dye mixture and transfer it to a spin filter.

77| Centrifuge for 10 minutes at 2240*g* and discard the filter.

CRITICAL STEP: Gel-dye mix should be kept for maximum 6 weeks within preparation and it is sufficient for 5 chips. Protect from light.

78| Pipette 9 µL of gel-dye mix in well G at the bottom of the well to prevent air bubbles.

79| To pressurise the gel-dye mix, place the chip in the priming station, lock it (click sound).

80| Position the syringe on 1 mL, set the timer for 1 min.

81| Press the plunger of the syringe down until it is held by the clip and time it.

82| After 1 min, release the clip and make sure it moves up to 0.3 mL. Pull the plunger to the 1 mL position.

83| Open the priming station and add 9 µL of gel-dye mix in the remaining **G** wells.

84| Load 5 µL of marker (green-capped tube) in all sample wells and ladder well.

85| Load 1 µL of ladder (yellow-capped tube) in the ladder well.

86| Pipette 1 µL of sample in each 11 sample wells.

87| Vortex the chip for 1 min in the IKA vortex mixer and start your run within 5 min.

88| Place the chip in the Bioanalyzer and close its lid carefully.

89| Check that the selection of dsDNA, High sensitivity DNA option is selected and the names of the samples are correctly filled in.

90| Start the run. A full run takes around 40min.

▪ EXPECTED OUTCOME

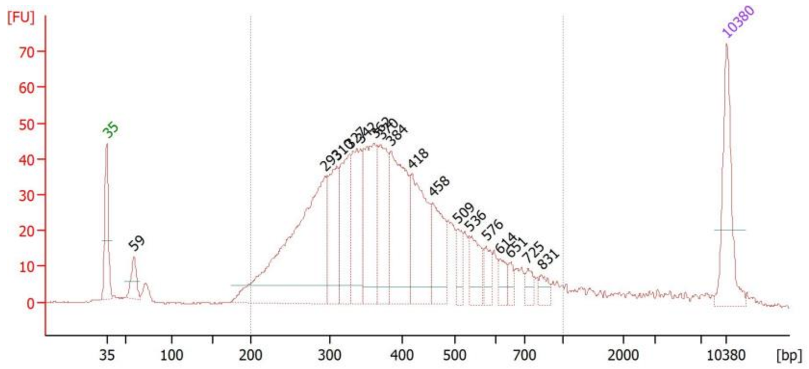

Size distribution varies according to the amount of DNA used, tagmentation time and extension time. Therefore, these can be adjusted to obtain a desired size distribution.

DNA library quantification by qPCR♦ TIMING: ≈1h

Quantification the libraries concentration by qPCR using the Universal KAPA library quantification kit.

91| Thaw all reagents from the kit: Master mix+ Primer premix, ROX and standards.

92| Prepare 1:1000 and 1:2000 dilutions of the libraries in 10 mM Tris-HCl, pH 8.0.

93| If the kit is used for the first time, add the 10X Primer Premix (1 mL) to the 2X KAPA SYBR® FAST qPCR Master Mix (5 mL) and mix. Add appropriate ROX (High or Low) depending on the qPCR instrument in your lab (0.2 mL).

94| Prepare your master mix accordingly for samples, six standards and appropriate replicates:

Component	Volume (µL)
2x qPCR Master Mix+ 10x Primer premix + 50x ROX	12.4
RNAse free-water	3.6

CRITICAL STEP: For more reproducible results we recommend duplicates or triplicates of sample and standards.

95| Pipette 16 µL of mix into each qPCR well.

96| Add 4 µL of standard or sample, accordingly.

97| Perform your qPCR with the following cycling conditions:

95°C for 5 minutes

95°C for 30 s|

60°C for 45 s| 35 cycles

OPTIONAL: Perform a melting curve analysis: 65-95°C

Pooling of the libraries into a 4 nM solution ♦ TIMING: ≈ 30 min

The 4 nM concentration aims for a higher cluster density.

98| Dilute the libraries aiming for 4 nM solution using the concentration obtained for the qPCR and the average size from the Bioanalyzer.

99| Make a pool taking into account the number of reads desired per sample.

CRITICAL STEP: Do not mix on the same pool samples with the same double indexing.
